# Terrie Fox Wetle Award (2021): Advancing multidisciplinary health services science: Developments in a Dementia-Focused Program of Research

**DOI:** 10.1093/geroni/igab046.1489

**Published:** 2021-12-17

**Authors:** Andrea Gilmore Bykovskyi

**Affiliations:** University of Wisconsin-Madison, Madison, Wisconsin, United States

## Abstract

The Terrie Fox Wetle Rising Star Award in health Services and Aging Research is an award named in honor of Fox Wetle, PhD, who is internationally recognized for her contributions to aging, public health, and health care research. The award recognizes health services researchers in early or middle-career phases who have made significant contributions that embody the value of multidisciplinary health services science and are likely to have a sustained, high impact on practice and research. This aware lecture will be presented by the 2021 Award Recipient, Andrea Gilmore-Bykovskyi, PhD, RN, and will highlight emergent findings and foci in her dementia-focused health services research program. In particular, the award lecture will discuss progress in investigating social and behavioral communication patterns among individuals with moderate to advanced dementia; and the role of temporally situated observational measures and inclusion of persons with dementia and their caregivers in this line of research. The lecture will conclude with a discussion of next steps for this area of investigation surrounding assessment of episodes of lucidity in advanced dementia; and considerations for strengthening progress in outcome evaluation among persons living with dementia through multidisciplinary and community-informed health services research.

